# The Relevance of DNA Methylation and Histone Modification in Periodontitis: A Scoping Review

**DOI:** 10.3390/cells11203211

**Published:** 2022-10-13

**Authors:** Andrew Liaw, Chun Liu, Sašo Ivanovski, Pingping Han

**Affiliations:** 1 Center for Oral-facial Regeneration, Rehabilitation and Reconstruction (COR3), The University of Queensland, Brisbane, QLD 4006, Australia; 2 School of Dentistry, Faculty of Health and Behavioural Sciences, The University of Queensland, Brisbane, QLD 4006, Australia

**Keywords:** epigenetics, epigenome, periodontitis, histone code, DNA methylation, epigenetic biomarker

## Abstract

*Background*: Periodontitis is a chronic inflammatory disease involving an interplay between bacteria, inflammation, host response genes, and environmental factors. The manifestation of epigenetic factors during periodontitis pathogenesis and periodontal inflammation is still not well understood, with limited reviews on histone modification with periodontitis management. This scoping review aims to evaluate current evidence of global and specific DNA methylation and histone modification in periodontitis and discuss the gaps and implications for future research and clinical practice. *Methods*: A scoping literature search of three electronic databases was performed in SCOPUS, MEDLINE (PubMed) and EMBASE. As epigenetics in periodontitis is an emerging research field, a scoping review was conducted to identify the extent of studies available and describe the overall context and applicability of these results. *Results*: Overall, 30 studies were evaluated, and the findings confirmed that epigenetic changes in periodontitis comprise specific modifications to DNA methylation patterns and histone proteins modification, which can either dampen or promote the inflammatory response to bacterial challenge. *Conclusions*: The plasticity of epigenetic modifications has implications for the future development of targeted epi-drugs and diagnostic tools in periodontitis. Such advances could be invaluable for the early detection and monitoring of susceptible individuals.

## 1. Introduction

Periodontitis is characterised by a disruption to the homeostatic integrity of the subgingival microbiota and host response, which promotes a self-sustained and reciprocal cycle of inflammation-driven tissue destruction and enhanced microbial dysbiosis [1,2,3,4]. This perturbation in tissue homeostasis and resulting periodontal destruction is facilitated by a variety of pro-inflammatory mediators, comprising interleukin (IL)1, IL6, IL8, interferon-gamma (IFNγ), tissue inhibitors of MMPs (TIMPs), and tumour necrosis factor-alpha (TNFα) [5,6]. A large body of evidence details the involvement of these cytokines in the innate immune response to bacterial challenge and tissue destruction.

It is well established that plaque biofilm is the key aetiological requirement for the initiation and progression of periodontitis [1,2,6,7], although in itself is insufficient for tissue destruction to occur, with a susceptible patient also necessary. Given the known effects of the environment and systemic health status on the epigenome and the established role of environmental exposures as determinants of disease susceptibility [7,8,9], epigenetic factors are particularly relevant in the pathobiology of periodontitis [10,11]. The term ‘epigenetics’, first coined in 1942, describes the reversible changes in gene expression patterns or cellular phenotypes that are not encoded in the DNA sequence [12]. Principal modes of epigenetic function include small non-coding RNAs, DNA methylation and histone modification [12,13]. For the purpose of this review, epigenetic modifications of DNA and histone proteins will be explored in the context of periodontitis.

**Figure 1:**
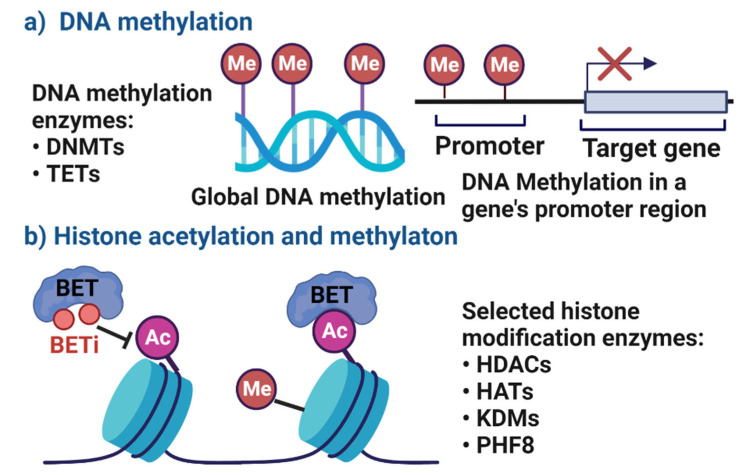
A schematic overview of epigenetic modifications to gene expression, regulated by DNA methylation enzymes (**a**) and histone modification enzymes (**b**). Ac, acetylation; DNMTs, DNA methyltransferases; HATs, histone acetyltransferases; HDACs, histone deacetylases; KDMs, histone demethylases; Me, methylation; PHF8, PHD finger protein 8; TETs, ten-eleven translocation family of enzymes. BET: bromodomain and extra-terminal proteins; BETi: BET inhibitors.

DNA methylation is a widely studied epigenetic mechanism due to its role in disease processes such as cancer, and normal biological cell function [12]. Gene-specific DNA methylation describes the analysis of the methylation status of particular genes, whilst global DNA methylation refers to the average methylation status across the whole genome (see Figure 1a). In mammalian cells, DNA methylation is replication-dependent and regulated by DNA methyltransferases (DNMTs) and ten-eleven translocation family of enzymes (TETs). Three major types of DNMTs (3a, 3b and 1) have been identified in humans, and each serves a different purpose. De novo DNMTs3a and 3b establish patterns of DNA methylation during early development and act after DNA replication. DNMT1 acts as a maintenance enzyme during DNA replication, responsible for reproducing unmethylated and methylated patterns to newly synthesized DNA strands [14]. As a whole, DNMTs facilitate the addition of methyl groups to the C5 position of cytosine residues of CpG dinucleotides. However, these CpG sites are usually rare and sparsely distributed in the genome, often being fully methylated. Alternatively, DNA methylation can also occur on CpG islands which are present in around 50% of human genes and mostly appear unmethylated in healthy tissue [15]. The unmethylated status of these CpG islands is often associated with transcriptionally active genes; thus, CpG methylation by DNMTs will result in transcriptional repression and gene silencing. This can cause unwarranted effects on key cellular processes, such as DNA repair, cell cycle regulation and expression of inflammatory mediators [12].

Histone modification can occur during post-translation stages through acetylation, deacetylation and methylation at amino acid tails (Figure 1b), directly altering selected regions on the chromatin structure [10]. Histone acetylation facilitated by histone acetyltransferases (HATs) involves the transfer of acetyl groups to lysine residues on the N-terminal tails of histones. This results in an open chromatin conformation, promoting transcription factor binding and increased gene transcription. In contrast, histone deacetylases (HDACs) remove acetyl groups from histones and condenses the chromatin structure into a closed conformation, repressing gene transcription [14]. In humans, there are 18 HDAC enzymes grouped into four main classes, of which Classes I and II are the most studied. Class I enzymes comprise HDACs1, 2, 3 and 8 which are confined to the nucleus, whilst Class II enzymes include two sub-groups HDACs 4, 5, 7 and 9 (class IIa) and HDACs 6 and 10 (class IIb), which can shuttle between the nucleus and cytoplasm, Class III enzymes include Sirtuins 1 to 7 and Class IV contains only HDAC11 [16]. The integrity of immune homeostasis relies on the delicate balance of acetylation and deacetylation activity, to prevent aberrant transcription of key immunomodulatory genes [10]. Lastly, histone methylation by lysine methyltransferases (KMTs) and lysine demethylases (KDMs) can activate or suppress gene expression. Such methylation events have an important role in the maintenance of cell cycle, DNA damage and stress response; however, their interaction with the inflammatory response is poorly understood [12].

The role of epigenetic factors in the context of periodontitis diagnosis is an emerging field [17,18,19,20,21,22,23], and the unique traits of epigenetic biomarkers could provide invaluable diagnostic and therapeutic insights into more predictable, precise and personalised disease management. The epigenetic factors, HDACs, DNA methylation and circular RNA, are shown to express in periodontal tissues [22], oral fluids such as saliva [20], and circulating small extracellular vesicles (sEVs) [17,18,19,20]. sEVs are a type of biological nanoparticles secreted by all cell types that are circulating in extracellular space (*i.e.,* biofluid or cell culture media), and containing genetic and epigenetic materials, protein, lipids and other biology molecules from their parental cells [17,19]. To date, few review papers outline the epigenetic mechanisms related to periodontitis, most of which are heavily focused on DNA methylation or non-coding RNA changes [9,22,24,25,26,27,28]. There are relatively limited reviews summarising HDACs in periodontitis pathogenesis and the use of HDAC inhibitors (HDACi) as adjunctive therapies for periodontitis treatment. Additionally, there is a lack of knowledge outlining the application of sEVs-associated epigenetics in periodontitis. Therefore, further review of the recent body of evidence is warranted, detailing the mediation of DNA, histone and sEVs epigenetic modulators in periodontal inflammation, and current developments in the utilization of histone deacetylase inhibitors in periodontitis management. This scoping review aimed to evaluate recent selective evidence of the epigenetic changes related to chemical modifications of DNA and chromatin structure in periodontitis and periodontal cells/tissues as well as potential histone epi-drugs for periodontitis treatment. Specifically, this review sought to answer the following questions:

In periodontally diseased tissues or oral biofluids, what are the observed alterations in sEVs, global DNA methylation or specific DNA methylation patterns of pro-inflammatory cytokines genes?

What is the current research regarding histone modification and its relevance in periodontal inflammation and dysbiosis?

## 2. Materials and Methods

### 2.1. Study Eligibility Criteria

Titles and abstracts from all retrieved records were screened for potentially eligible studies by one reviewer (A.L.). For inclusion in the review, the studies were required to meet the following eligibility criteria:

- Patients with periodontitis;
- Gingival biopsies, gingival crevicular fluid or saliva samples;
- Retrospective and prospective studies of the observational, case–control, cohort, or randomised controlled trial design;
- English language.

The exclusion criteria were as follows:

- In vitro and in vivo studies (for DNA methylation—refer to 2.3);
- Case reports;
- Literature reviews;
- Diagnoses of aggressive periodontitis;
- Human blood or mouth rinse samples.

### 2.2. Search Strategy

A search strategy, using Boolean operators was employed to identify papers using MesH, keywords and other free terms: (periodont* OR “periodontal infection”) AND (“DNA methylation”) OR (“Histone modification” OR “histone acetyl*” OR “histone methyl*” OR “histone deacetyl*” OR “histone phosphorylation”) OR (“epigen* OR “epigenetic biomarker” OR “epigenomic biomarker”).

The search was conducted using three electronic databases: SCOPUS, MEDLINE (PubMed) and EMBASE. No filter for the date was placed to ensure that all relevant articles were included. In addition, a manual search for relevant selective studies from the past ten years was performed for five selected journals: *Journal of Clinical Periodontology*, *Journal of Periodontology*, *Journal of Dental Research*, *Journal of Periodontal Research* and *Periodontology 2000*.

### 2.3. Study Selection Strategy

Following a screening of titles and abstracts, potentially relevant studies were retrieved, as well as the full text for studies where eligibility was uncertain based on the title and abstract alone. All full-text studies were independently assessed in detail to determine whether they fulfilled the pre-specified inclusion/exclusion criteria. Studies that met the selection criteria were processed for data extraction. The reference lists of these selected studies were also hand-searched for any additional relevant articles. To address the clinical context of the first review question, clinical studies of specific and global DNA methylation in humans were exclusively selected. For the second review question, only one human study describing histone modification was retrieved from the preliminary search; therefore, to broaden the discussion for this focus area only, the inclusion criterion was revised to selectively include in vivo and in vitro studies.

## 3. Results

### 3.1. Literature Search and Screening Process

**Figure 2:**
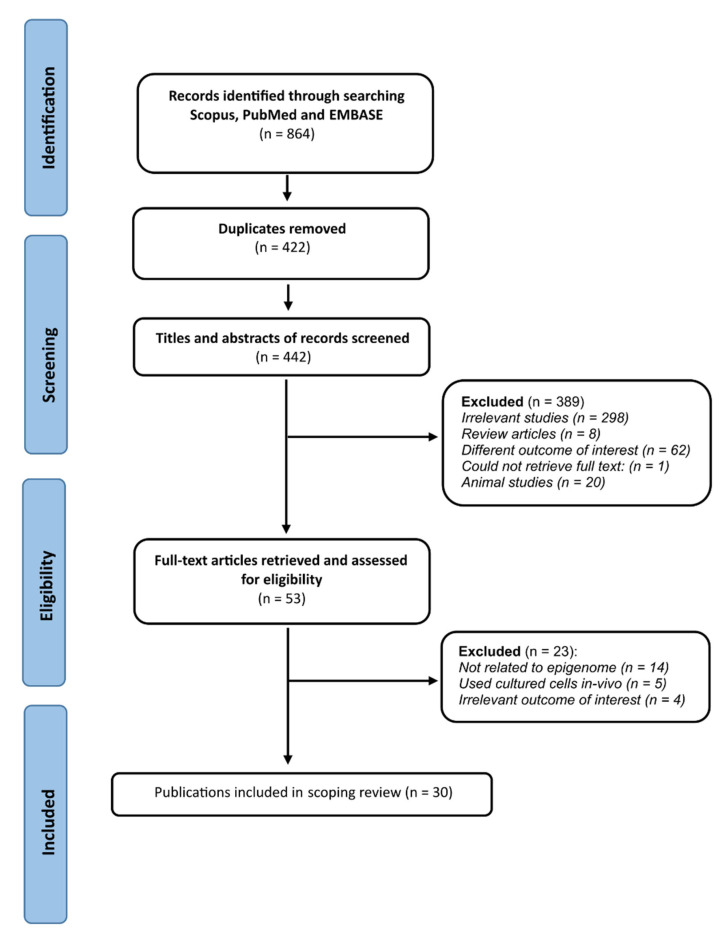
Preferred Reporting Items for Systematic Reviews and Meta-Analysis (PRISMA) flow diagram detailing study screening and selection.

The initial electronic and manual search yielded a total of 864 abstracts. In the preliminary stage of the study selection process, 422 records were excluded after removing duplicates, and a further 389 studies were excluded after the evaluation of titles and abstracts (Figure 2). The complete full-text articles of the remaining 53 studies were further assessed for eligibility according to pre-specified inclusion and exclusion criteria. A total of 30 studies were deemed eligible for inclusion in this scoping review (Figure 2).

### 3.2. Study Characteristics

A summary of included studies’ characteristics is described in Table 1, Table 2 and Table 3. Briefly, 15 studies were of cross-sectional design, 14 studies of in vitro experimentation, and 1 study involved a case-control sample. All studies were published in English. Thirteen studies assessed the epigenetic modifications using gingival biopsies, two studies assessed alveolar bone and soft tissue in mice, two studies analysed oral biofluid including whole unstimulated saliva and gingival crevicular fluid, and the remaining studies cultured cells in vitro, including gingival epithelial cells, PDL stem cells, keratinocytes, monocytes and osteoclast progenitors.

### 3.3. Changes in DNA Methylation Patterns and Gene Expression Profiles in Periodontitis Patients

This review identified 15 relevant studies analysing the DNA methylation status of immune-related genes implicated in periodontitis. Of these studies, there were two longitudinal studies [29,30], and the remaining 13 publications were of cross-sectional design [19,31,32,33,34,35,36,37,38,39,40,41,42]. All publications in Table 1 were critically assessed to explore correlations between methylation status, transcription levels and periodontitis.

De Souza and co-workers conducted a high-throughput methylation microarray (Illumina) on gingival tissues from 12 periodontitis patients and 11 age-matched healthy individuals [37]. The results showed that significant differences in methylation variation in pro-inflammatory genes exist between healthy and periodontitis tissues, including a wide range of cytokines, cytokine receptors, growth factors, transcription factors and cell membrane proteins. These methylation changes are inversely correlated with their respective levels of mRNA expression. More recently, Han and colleagues observed a significantly higher methylation status of global 5-methylcytosine and N6-Methyladenosine in salivary extracellular vesicles in periodontitis patients compared with health [19].

#### 3.3.1. Changes in DNA Methylation Patterns of Cytokine-Encoding Genes

The comparison of human gingival biopsies from periodontitis and healthy tissues have demonstrated distinct hypermethylation patterns of TIMP1 [35] and TNFα [31] promoter regions in periodontitis. Moreover, TIMP1 hypermethylation correlated with disease severity, with positive associations with probing depths and negative correlations with bleeding on probing [35].

Zhang et al. [33] reported a hypomethylated profile of IFNγ promoter regions, concomitant with a 1.96-fold increase in IFNγ levels in periodontally diseased tissues compared with their healthy counterpart. In contrast, Asa’ad et al. [29] and Viana et al. [42] reported no significant differences in methylation status of IFNγ and IL10 gene promoter regions in periodontitis versus healthy tissues.

Other studies have explored the methylation levels of other potential targets, such as IL6 [34] and tet-methylcytosine dioxygenase 2 (TET2) enzymes [36], and observed partial methylation of periodontitis tissues with increased levels of gene expression.

#### 3.3.2. Changes in DNA Methylation Patterns of Cellular Signalling Genes

Genes related to cellular signalling are another focused target of studies on DNA methylation in periodontitis. Differential DNA methylation at the promoter regions of 12 genes (DCC, KCNA3, KCNA2, RIMS2, HOXB7, PNOC, IRX1, JSRP1, TBX1, OPCML, CECR1, SCN4B) has been detected between periodontitis and clinically healthy tissues [41].

Cyclooxygenase-2 (COX-2) gene promoter (PTGS2), a key regulatory gate for prostaglandin E2 (PGE2), appears to be hypermethylated in periodontitis gingival tissues [29,32], with methylation levels of up to 5-fold higher than healthy tissue. This trend was inversely correlated with decreased PTGS2 gene transcription and may serve as an intrinsic protective mechanism to prevent the uncontrolled breakdown of the periodontium.

A study conducted by de Faria Amormino and co-workers [39] observed hypermethylated TLR2 gene profiles in gingival biopsies of chronic periodontitis patients, correlated with probing depth parameters and quantity of inflammatory cells. This was associated with repressed TLR2 gene expression which may facilitate a chronic persistence of bacterial perturbation within the periodontal tissues. Similar findings were reported by De Oliveira and colleagues [38], observing trends for hypermethylation in TLR2 genes and significant hypomethylation of TLR4 gene profiles in periodontitis tissues compared with healthy samples. These effects on TLR2 and TLR4 gene expression may have significant implications on the integrity of the host-microbe crosstalk and in part, may contribute to dysbiosis exacerbation.

More recently, Azevedo and co-workers [40] screened a selection of targets and showed hypomethylation of signal transducer and activator of transcription 5 (STAT5) genes in periodontitis gingival tissues compared with their healthy counterparts. This suggested that loss of methylation in the promoter region of the STAT5 gene may occur during the development of periodontitis. This hypomethylation pattern has been associated with upregulation in its respective gene expression, which may promote the induction and survival of specific cell lines in periodontal lesions, regulate T-cell differentiation, and regulate the inflammatory environment within the periodontal pocket [43].

#### 3.3.3. Changes in DNA Methylation Patterns following Periodontal Therapy

Asa’ad and colleagues [29] explored the methylation levels and gene expression pattern of TNFα, IFNγ and COX-2 in gingival biopsies over two months following non-surgical periodontal therapy. They concluded that treatment could reset and significantly reduce COX-2 methylation to a level comparable to health; however, this had little effect on TNFα and IFNγ. The authors postulated that for such mediators, their epigenetic modifications might be locally sustained even after the reduction in periodontal inflammation.

Similarly, a study by Andia et al. reported after 3 months of periodontal treatment and resolution of inflammation, the methylation profiles of SOCS1, SOCS3 and LINE-1 return to a level that is comparable with health [30].

#### 3.3.4. Changes of sEVs-Associated Epigenetic Markers in Periodontitis

Small extracellular vesicles (sEVs) are a new class of potential liquid biopsy that are biolayered lipid nanoparticles carrying a cargo of protein, epigenetic/genetic material, lipids and metabolites from their parent cells [21,44]. The use of sEVs from oral biofluids in diagnosing periodontitis is an emerging field and has been recently reviewed by the authors [17]. Our pilot studies showed that periodontitis salivary sEVs contain DNA methylation of pro-inflammatory cytokines genes in gingivitis [23], sEVs-microRNAs [18] and global DNA methylation [19]. Of note, compared with whole unstimulated saliva, salivary sEVs-associated global 5mC hypermethyaltion and microRNAs (hsa-miR-140-5p, hsa-miR-146a-5p and hsa-miR-628-5p) can distinguish periodontitis and healthy patients [18,19]. Furthermore, sEVs also contain circular RNA (i.e., subtype of non-coding RNAs) that are involved in periodontitis pathogenesis as recently reviewed by the authors [22]). However, this requires more studies in the field to further explore the sEVs epigenetic markers in diagnosing and treating periodontitis.

#### 3.3.5. Conclusions from the Included Studies

Overall, the findings from these studies highlight the local alterations of DNA methylation patterns in periodontally diseased tissues versus health. These epigenetic modifications to gene promoter regions and their resulting effects on gene expression can work to activate a series of downstream mechanisms that minimise uncontrolled periodontal tissue destruction.

### 3.4. Histone Modification and Its Relevance in Periodontal Inflammation

This review identified ten relevant studies analysing post-translational histone modifications implicated in chronic periodontitis, and seven relevant studies focusing on HDACi for treating periodontitis. Of these studies, there was only one cross-sectional study [45], two in vivo studies [46,47], and the remaining publications were in vitro [48,49,50,51,52,53,54,55,56,57,58,59]. All publications in Table 2 and Table 3 were critically assessed to explore correlations between histone modification and the periodontal inflammatory response, along with the effects of various novel HDACi on human in vitro cells.

#### 3.4.1. The Role of Histone Deacetylases, Methylases and Demethylases in Periodontal Inflammation

Most of the studies investigating histone modification stimulated human epithelial or monocytic cells in vitro to *P. gingivalis* and/or Fusobacterium nucleatum LPS. This perturbation to the balance of histone acetylation/deacetylation and methylation/demethylation results in disruption of epigenetic regulation of key regulatory genes and differential expression of pro-inflammatory mediators in periodontitis.

Histone methylation serves to maintain both active and suppressed states of gene expression, depending on the sites of methylation. Yin and colleagues [48] assessed the methylation of Lys4 of histone 3 (H3K4), which is associated with transcriptionally active chromatin. When gingival epithelial cells were exposed to *P. gingivalis* or *F. nucleatum* for 24 h, there were decreased protein concentrations and differential induction of H3K4 methylation compared with unstimulated cells. Similarly, when PDL stem cells were exposed to *P. gingivalis* LPS, H3K27me3 and H3K4me3 expression were increased on extracellular matrix and osteogenesis lineage genes, and inflammatory response genes, respectively [53].

There is growing evidence supporting the role of dysregulated epigenetic modulation of histone demethylases (KDMs) in the inflammatory response, particularly in cancer and viral infections. In the context of periodontitis, substrates of KDMs have been linked to the regulation of inflammatory and osteoclastic processes. Kirkpatrick et al. observed a significant increase in both KDM4B and KDM4E abundance in periodontally diseased connective tissue compared with healthy tissue [52]. Other subtypes of KDMs including KDM3C [54] and PHF8 [55] have demonstrated protective effects in regulating pro-inflammatory cytokine signalling and differentiation of osteogenic precursors, which appears to be suppressed with exposure to *P. gingivalis* LPS.

Histone acetylation and deacetylation have been implicated in epigenetic modifications of gene expression in periodontitis. Inflammation in the periodontal microenvironment stimulated downregulation of histone acetyltransferase GCN5, resulting in decreased osteogenic differentiation of PDL stem cells [49]. In experimental models where oral epithelial cells were exposed to LPS from *P. gingivalis* and *F. nucleatum*, abrupt, short-lived acetylation of histone H3 and accumulation of p300/CBP was observed [50]. The authors suggested that targeting the disruption to p300/CBP could restore tissue homeostasis through regulation of pro-inflammatory cytokine activity, and cellular differentiation, proliferation, and apoptosis.

Histone deacetylases are epigenetic markers of increasing interest, due to their potential for therapeutic targets in bone destructive diseases. Currently, there is a paucity of data on the alterations in HDAC expression in periodontal inflammatory conditions. In [48], significantly decreased *HDAC1* and *HDAC2* gene expression was observed in gingival epithelial cells and keratinocytes challenged by *P. gingivalis* and *F. nucleatum*. In monocytes stimulated by TNFα, Algate et al. observed increased expression in Class I and II HDACs [59]. *HDAC9* RNA expression levels appear to be significantly increased in PDL stem cells under inflammatory conditions driven by periodontitis or exposure to TNFα, which impaired osteogenic capacity in vitro [51].

#### 3.4.2. Changes in Histone Deacetylase Expression in Periodontitis versus Health

Cantley and co-workers [45] compared gingival biopsies of chronic periodontitis patients with their healthy counterparts. An elevated mRNA expression of Class I *HDAC1* and *8* and Class II *HDAC5* and *9* was found in periodontally diseased samples. Moreover, immunohistochemical staining demonstrated expression of HDAC1 by CD3- and TNF-α positive cells, indicating that such cells may be important additional targets to suppress inflammation, suggesting that HDACs modification may be involved in periodontitis pathogenesis. However, the implications of these higher levels of expression in periodontitis are unclear. More studies are required to warrant the role of HDACs in periodontal tissues and oral biofluids.

#### 3.4.3. The Effects of Histone Deacetylase Inhibitors on the Periodontal Inflammatory Response

The modulation of HDACs using inhibitors is an emerging treatment for cancer, neurodegenerative diseases, autoimmune disorders and viral infections. This is largely related to its ability to upregulate cell-cycle inhibitors, downregulate immune stimulators, and suppress pro-inflammatory cytokines and osteoclast activity. Novel types of HDACi have progressed to clinical trials, with the vast majority targeting multiple HDACs in both classes I and II (i.e., ‘pan’ inhibitors). In the context of periodontitis, their application is limited to in vitro and in vivo experimental models of inflammatory bone loss.

Algate et al. investigated novel selective inhibitors of specific HDACs classes, utilising the stimulatory effects of TNF to induce the inflammatory response in human monocytic cells [59]. Treatment with Class I HDAC1i and HDAC2i demonstrated an anti-inflammatory effect through a dose-dependent suppression of IL-1β, TNF, MIP-1α and MCP-1 mRNA expression. Moreover, inhibition of osteoclastogenesis was observed through the downregulation of TRAF-6 and NFATc1, which subsequently reduced the expression of Cath K, DC-STAMP and TRAP osteoclast genes. Interestingly, no effects were seen with Class II HDAC5i and HDAC7i [59]; however, HDAC-9i restored the osteogenic differentiation capacity of inflamed PDL stem cells to a level comparable with their healthy counterparts [51].

Although these studies highlight that HDACi targeting single classes could reduce osteoclastic bone resorption, Cantley and colleagues [56] explored the efficacy of a broader spectrum HDACi (targeting Class I and II), known as 1179.4b. In their initial in vitro study, 1179.4b produced a strong suppression of osteoclast transcription factors NFATc1 and OSCAR and a substantial reduction in CTR and TRAP gene expression. Such changes corresponded with a marked inhibition of bone resorption in vitro. These bone-protective effects were further confirmed in periodontitis-induced mice treated with 1mg/kg/d of 1179.4b, observing significant reductions in osteoclasts and alveolar bone loss [46]. However, interestingly, no reductions in inflammation of the gingival tissues occurred. This likely suggests that the 1179.4b exerts its mechanistic effects directly on bone tissue rather than indirectly through suppression of inflammation. When comparing the bone resorption effects of 1179.4b against a selective inhibitor of HDAC Class I (MS-275), MS-275 appeared to reduce gingival inflammation; however, it had no observed effect on bone loss.

Further investigations of combined Class I and II HDACi compounds, such as Trichostatin A (TSA), were conducted by Huynh and colleagues [47,57]. When pre-treating PDL stem cells with TSA, osteogenic differentiation of PDL stem cells were induced and osteoblast-related gene expression increased. This subsequently enhanced mineral deposition and bone regeneration in vivo of mouse calvaria defects.

Lagosz et al. detailed the effects of treating healthy and periodontally diseased gingival fibroblasts with pan-HDACi and selective HDAC3/6i. Treatment of both healthy and periodontally diseased gingival fibroblasts with pan-HDACi or HDAC3/6i prior to *P. gingivalis* infection significantly reduced the induction of a subset of inflammatory mediators (CCL2, CCL5, CXCL10, IL1B, COX2 and MMP3) implicated in periodontitis [58].

#### 3.4.4. The Effect of Bromodomain and Extra-Terminal Proteins Inhibitors in Periodontitis

Recent studies in the field of epi-drugs have evaluated small molecule inhibitors known as bromodomain and extra-terminal motif proteins inhibitors (BETi) that reversibly bind the bromodomains and prevent protein–protein interaction between BET proteins and acetylated histones [60]. For example, BETi, I-BET151 and JQ1, were shown to suppress inflammatory gene expression (IL6, IL1β and CCL2) in gingival fibroblasts and telomerase-immortalized gingival keratinocyte cell line (GEC line TIGK) after *P. gingivalis* stimulation [61]. Systemic administration of BETi-JQ1 in a murine periodontitis model showed a significantly inhibited inflammatory cytokine expression in diseased gingival tissues and alleviated alveolar bone loss through osteoclast reduction in periodontal tissues [62]. Moreover, the use of Apabetalone inhibited *P. gingivalis* LPS-induced macrophage M1 polarization via regulating miR-130a/STAT3 axis [63]. Overall, current evidence highlights the potential of BETi to regulate key inflammatory mediators in periodontitis and warrants further clinical translation to evaluate their efficacy as periodontal treatment adjuncts.

#### 3.4.5. Conclusions from the Included Studies

It is difficult to translate the current in vitro and in vivo findings into the context of human periodontitis, as existing models fail to address that periodontitis is associated with a complex community of oral bacterial species, including commensals, rather than a single dominant species. Therefore, the role of various KDMs, histone acetylases and HDACs in the response to bacterial challenge may ultimately differ depending on the individual specific pathogenicity of their microbial profile. Nonetheless, one cannot refute the evidence of epigenetic effects on histone modification in the periodontal inflammatory response, which has pioneered the advancement of precision medicine and ‘epi-drugs’.

The focus of HDACi within periodontitis, albeit confined to animal and experimental studies, has mostly shown that such compounds can dampen the exaggerated inflammatory response to plaque biofilm, and potentially suppress osteoclast activity. Furthermore, there may be some redundancy in targeting specific HDACs in periodontitis, as a combination of HDAC classes may need to be inhibited for more potent effects. Nonetheless, these compounds show promising clinical benefits as an adjunct to conventional periodontal therapy.

## 4. Discussion and Conclusions

**Figure 3:**
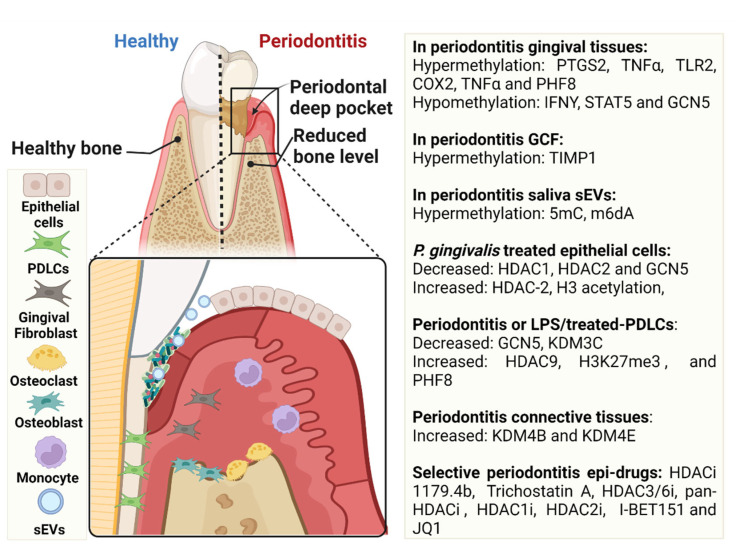
Summary of key epigenetic modulators of DNA methylation and HDACs during periodontitis pathogenesis and periodontal cells under inflammatory microenvironment.

This review identified the current evidence relating to epigenetic modifications in periodontitis, comprising specific modifications to DNA methylation patterns and histone proteins. Hypermethylation and hypomethylation of various gene promoter regions associated with pro-inflammatory cytokines, and upregulation and downregulation of HDAC activity in gingival tissues, GCF, saliva sEVs and cultured periodontal cells under inflammatory conditions have been well demonstrated. These epigenetic alterations can either dampen or promote the inflammatory response to bacterial challenge (see Figure 3).

### 4.1. Implications of the Findings for Research

Most existing studies utilise a cross-sectional design, providing a snapshot of disease activity at a single time point. Given that periodontitis is a dynamic inflammatory disease fuelled by dysbiosis, with periods of remission and exacerbation, cross-sectional epigenetic studies provide limited insight into disease onset and progression over time from the host perspective [64]. Rather, future longitudinal studies are warranted to monitor epigenetic modifications in periodontitis over time and identify susceptible and non-responding individuals. Large-scale epidemiological epigenome-wide association studies may also have merit, preferably through widespread screening of DNA methylation markers to reduce data extraction errors [35].

Whilst addressing the second review question, a gap in the literature was identified, with no longitudinal human studies conducted on histone modification changes in the periodontal tissues. Preliminary evidence from current in vitro and in vivo studies has demonstrated marked histone modulatory changes in response to periodontopathic bacterial challenge (see Figure 3). However, their use of single pathogen-induced LPS models inherently has severe limitations in applicability to humans; therefore, future research needs to be carried out in the clinical setting. This could entail the long-term follow-up of histone modifications in treated and untreated patients with various disease severities. Such study designs could broaden our current understanding of periodontitis aetiology and susceptibility.

### 4.2. Implications of the Findings for Clinical Practice

Research into epigenetic biomarkers may facilitate a more detailed understanding of the interactions between an individual, their genes and the environment, which predispose them to periodontitis. This forms the basis for precision medicine, where epigenetics could be used as a reliable tool to identify why certain patients with the same clinical phenotype do not respond to conventional periodontal treatment. Most of the existing human studies exploring this topic have collected gingival biopsies from periodontitis patients; however, this may deter patient acceptance and invalidate sample reproducibility [65]. Other less invasive and easily accessible DNA sources, such as gingival crevicular fluid and saliva, could be considered clinically feasible diagnostic tools for targeted epigenetic analysis [66]. It is worth noting that applying DNA methylation and histone modification in the clinic is still at its infancy, due to limited understandings of epigenetic mechanisms in periodontitis pathogenesis. More studies are required to validate the role of epigenetics in periodontitis disease management.

Although still an emerging field, understanding periodontitis-induced epigenetic changes in DNA methylation and/or histone modifications could point towards new targeted drug developments in periodontal management. Furthermore, the plasticity of epigenetic modifications has driven increased interest in the development of targeted epi-drugs which could take advantage of this reversible state to ultimately restore a normal epigenetic landscape [33]. Recent studies evaluating HDACi have demonstrated promising results in the field of medicine, with implications for periodontitis treatment using 1179.4b, TSA, HDAC3/6i, pan-HDACi, HDAC1i and HDAC2i (see Figure 3). With its unique ability to target bone remodelling, HDACi therapeutics could serve as a significant advance in personalised periodontal treatment approaches.

## Figures and Tables

**Table 1:** Summary of selected publications included for analysis of the periodontitis-DNA methylation relationship.

Author/Year	Global or Target Genes	Tissue/Cell Type	Sample Size	Case Definitions	Key Epigenetic Events and Conclusions
Kim et al., 2021	DEGs (RNA sequencing, DNA methylation profiling) Global and specific gene DNA methylation	Gingival biopsies	12 patients with Stage III periodontitis (n = 12), gingivitis (n = 12), periodontal health (n = 12)	Stage III periodontitis: PD ≥ 5 mm with CAL ≥ 3 mm, radiographic bone loss beyond the coronal third of the root, and BoP. Gingivitis: PD ≤ 3 mm, no CAL, and presence of BoP. Healthy: PD ≤3 mm, no CAL, and no BoP.	Twelve DEGs between periodontitis tissues and clinically healthy tissues (DCC, KCNA3, KCNA2, RIMS2, HOXB7, PNOC, IRX1, JSRP1, TBX1, OPCML, CECR1, SCN4B) were differentially methylated between the two phenotypes.
Han et al., 2021	5mC, 5hmC, m6dA (DNA methylation, qPCR) Global DNA methylation	Unstimulated whole saliva (salivary extracellular vesicles)	Periodontal health (n = 7), gingivitis (n = 7) and periodontitis (n = 8)	Healthy: no periodontal disease history; PPD < 3 mm; BOP < 15 % sites. Gingivitis: no periodontal pocket, PPD < 3 mm; BOP > 30 % sites. Stage III/IV periodontitis: > 30% of the sites with PPD ≥ 3 mm and BOP, at least five sites with PPD ≥ 5mm on at least three non-adjacent teeth.	Global 5 mC and m6dA methylation was significantly increased in periodontitis salivary extracellular vesicles compared with health.
Azevedo et al., 2020	MALT1, LTB and STAT5 (DNA methylation, PCR) specific gene DNA methylation	Gingival biopsies	Periodontitis (n = 20) and healthy individuals (n = 20).	Periodontitis group: >30% of teeth with interproximal CAL ≥ 5 mm, radiographic bone loss extending the apical third of the roots, loss ≥ 5 teeth due to periodontitis, PD ≥ 7 mm. Control group: PD < 4 mm during tooth extraction for orthodontic treatment or of third molar and crown lengthening	Overall, all genes in the periodontitis group showed an unmethylated profile. In comparison with the control group, STAT5 showed a hypomethylated profile in periodontitis tissues.
Li et al., 2018	MMP-9 and TIMP-1 (Sodium bisulfite modification, PCR, pyrosequencing) Global and specific gene DNA methylation	Gingival crevicular fluid	Chronic periodontitis patients (n = 88) and healthy controls (n = 15)	Severe periodontitis group (n = 27) had PD (6.88 ± 0.83 mm) and CAL > 7 mm with bleeding upon probing each tooth. Moderate periodontitis (n = 29) had PD (5.78 ± 1.09 mm) and CAL <6 and 4 mm with bleeding upon probing. Mild periodontitis group (n = 32) had PD (2.45 ± 0.21 mm) and CAL<3 mm with bleeding upon probing. Subjects in the control group (n = 15) PD < 2 mm and no CAL, bleeding on probing, or radiographic bone loss.	TIMP-1 methylation levels gradually increased with periodontitis severity, with the highest methylation levels in those with severe periodontitis. Compared to healthy samples, periodontitis groups had a higher average methylation level. No significant differences in MMP-9 methylation levels between healthy and periodontitis patients were found.
Asa’ad et al., 2017	TNF-α, IFN-γ, COX-2 (DNA methylation, PCR) Specific gene DNA methylation	Gingival biopsies	Periodontal health (n = 10) moderate chronic periodontitis (n = 10)	Healthy group: PPD ≤ 3 mm, without any signs of tooth mobility, BOP or CAL. Periodontitis group: Patients in this group displayed a minimum of a single “healthy site” (PPD < 4 mm without signs of bleeding on probing, mobility or inflammation) and a periodontitis site (PPD ≥ 5 mm with bleeding on probing at baseline).	COX-2, TNF-α and IFN- γ were hypermethylated in periodontitis tissues compared with healthy patients. Following periodontal treatment, decreased COX-2, TNF-α, and unchanged IFN- γ methylation levels were found post-periodontal therapy
Larsson et al., 2016	TET2 enzyme (qPCR, DNA methylation) Specific gene DNA methylation	Gingival biopsies	Generalized severe chronic periodontitis (n = 21) and gingivitis (n = 17)	Periodontitis: bone loss ≥ 50%, PPD) ≥ 6 mm and bleeding on probing (BoP) Gingivitis: no signs of bone loss, PPD ≤ 4 mm and BoP	There was a significantly larger proportion of TET2-positive cells in periodontitis lesions than in gingivitis lesions. Expression of TET2 and IDH genes did not differ between gingivitis and periodontitis
Andia et al., 2015	SOCS1, SOCS3 and LINE-1 (bisulfite modification, Methylation-specific high-resolution melting analysis) Global and specific gene DNA methylation	Gingival biopsies	Healthy (n = 10), periodontitis (n = 10)	Healthy: no BOP, no CAL, FMB S ≤ 25%, and absence of tooth mobility. Periodontitis: CAL ≥ 5 mm, and radiographic bone loss in at least eight teeth and bleeding following pocket probing	The methylation profiles of SOCS1, SOCS3 and LINE-1 genes were similar between controlled periodontitis and healthy patients in epithelial and connective tissues.
De Souza et al., 2014	Immune-inflammatory, cell-cycle and stable genes (Genome-wide methylation chip, gene information) Global and specific gene DNA methylation	Gingival biopsies	Chronic periodontitis (n = 12) and healthy age- and gender-matched control individuals (n = 11)	Periodontitis: more than 3 CAL≥ 5 mm and bleeding on probing. Control group: no bleeding on probing, and all teeth with CAL ≤ 3.5 mm	Variations in methylation status between healthy and periodontitis cases were more significant in genes related to the immune system process, which showed a higher tendency for DNA unmethylation. This methylation pattern was inversely proportional to the mRNA levels of the immune genes during periodontitis.
Stefani et al., 2013	IL-6 (Bisulfite modification, MSP) Global and specific gene DNA methylation	Gingival biopsies	Periodontitis (n = 21) and control group (n = 21).	Periodontitis: PD ≥ 4 mm, gingival bleeding, CAL ≥ 3 mm Control group: absence of signs of periodontal disease, PD<4mm	No difference in methylation pattern between groups were identified. The IL-6 expression was higher in periodontitis than control group. No significant differences were observed between moderate and severe periodontitis.
De Faria Amormino et al., 2013	TLR2 (DNA methylation, RT-qPCR) Specific gene DNA methylation	Gingival biopsies	Periodontitis (n = 20) and control samples (n = 20)	Periodontitis group: a PD of ≥4 mm and CAL ≥ 3 mm and lesions distributed on more than two quadrants were included in this group. Control: absence of CAL, no sites with PD > 3 mm and absence of bleeding.	TLR2 gene had a hypermethylated profile in chronic periodontitis tissues.
Zhang et al., 2013	TNF-α (Bisulfite-Specific PCR, Pyrosequencing, RT PCR) Global and specific gene DNA methylation	Gingival biopsies from healthy or periodontitis patient	Chronic periodontitis (n = 17) (CP) and periodontally healthy individuals (n = 18). Another 11 individuals participated in an experimentally induced gingivitis study.	Periodontitis: at least 5 mm PD and radiographic evidence of alveolar bone loss (ABL). Health: a PD measurement of ≤4 mm, no BOP, and no evidence of radiographic ABL.	Two CpG sites within the TNF-α promoter (at −163 and −161 bp) displayed hypermethylation in severe chronic periodontitis samples compared with periodontal health. Samples from patients with experimental gingivitis showed no significant differences in the TNF-α promoter methylation patterns
Viana et al., 2011	IFN-γ and IL-10 (MSP, BSP, bisulfite modification) Specific gene DNA methylation	Gingival biopsies	Periodontitis group (n = 18), control group (n = 16).	Control group: no CAL, no bleeding on probing and PD ≤ 3 mm. Periodontitis: PD ≥ 4 mm, bleeding on probing and with extensive radiographic bone loss.	Methylation status of IFN-γ and IL-10 was not significantly different in chronic periodontitis compared with healthy gingival tissues
De Oliveira et al., 2011	TLR2 and TLR4 (PCR, DNA methylation) Specific gene DNA methylation	Gingival biopsies	Healthy controls (n = 11), periodontitis smokers (n = 11), periodontitis non-smokers (n = 12).	Healthy controls: absence of CAL and no sites with PD >3 mm. Periodontitis smokers (n = 11): Subjects with at least three teeth exhibiting sites ≥5 mm CAL in at least two different quadrants and had consumed five cigarettes per day for at least 5 years. Periodontitis non-smokers (n = 12): Subjects with at least three teeth exhibiting sites ≥5 mm CAL in at least two different quadrants.	In periodontitis tissue, a pattern of unmethylation was observed in the TLR4 gene, whereas a trend towards methylation was observed in the Hha I CpG site in the TLR2 gene; however, overall, there was no difference between healthy and periodontitis groups.
Zhang et al., 2010	PTGS2 (Bisulphite specific PCR, RT PCR and sequencing) Specific gene DNA methylation	Gingival biopsies	16 participants, aged between 18 and 65 yrs. Inflamed (n = 10), non-inflamed (n = 6).	Periodontitis: PD>5 mm, bleeding on probing, and radiographic evidence of localized bone loss. Health: probing depth measurements of ≤4 mm at all 4 inter-proximal probing sites and no bleeding on probing.	The overall methylation level of the PTGS2 promoter in chronically inflamed gingival tissues was 5.06-fold higher than the methylation level exhibited in non-inflamed gingival tissues.
Zhang et al., 2010	IFN-γ (DNA methylation, Bisulphite specific PCR and pyrosequencing) Global and specific gene DNA methylation	Gingival biopsies	Periodontally healthy sites (n = 23), Experimentally induced gingivitis sites (n = 12) and chronic periodontitis sites (n = 12).	Health: PD<4mm, no BOP, no evidence of radiographic bone loss Periodontitis: Those biopsied tissues were from sites exhibiting PDs of ≥5 mm, BOP and radiographic evidence of localized bone loss.	All six CpG sites within the IFN-γ promoter had significantly lower methylation levels in periodontitis than in periodontal health. This hypomethylation profile correlated with a 1.96-fold increase in IFN-γ transcription in periodontitis than in tissues with periodontal health.

Abbreviations: COX-2, cyclooxygenase-2; Differentially expressed genes, (DEGs); IL-6, interleukin-6; IFN-γ, interferon-gamma; LTB, lymphotoxin beta; MALT1, mucosa-associated lymphoid tissue lymphoma translocation protein 1; MMP-9, matrix metalloproteinase-9; PTGS2, prostaglandin-endoperoxide synthase-2; STAT5, signal transducer and activator of transcription 5; TET2, tet methylcytosine dioxygenase 2; TIMP-1, tissue inhibitor of metalloproteinases; TLR, Toll-like receptor; TNF-α tumour necrosis factor alpha; 5-methylcytosine, 5mC; 5-hydroxymethylcytosine, 5hmC; N6-Methyladenosine, m6dA.

**Table 2:** Summary of representative publications included for analysis of the periodontitis-histone modification relationship.

Author/Year	Target Genes	Tissue/Cell Type	Sample Size	Case Definitions	Epigenetic Events and Conclusions
Liu et al. 2021	Histone demethylase *PHF8* (Real-time PCR for mRNA expression analysis)	PDL stem cells	Not reported	Not reported	mRNA expression levels of *PHF8* were significantly upregulated during the osteogenic differentiation of PDL stem cells, and exposure to *P. gingvalis*-induced lipopolysaccharide (LPS) inhibited PHF8 expression.
Algate et al. 2020	*HDAC1, HDAC2, HDAC5* (2−ΔCT method)	Monocytic cells	Not reported	Not reported	Stimulation of monocytes with TNFα for 24 h resulted in increased expression of *HDAC1* and *HDAC2*.
Lee et al. 2019	KDM3C (RT-qPCR)	Monocytic cells	Not reported	Not reported	Cells challenged by *P. gingivalis* LPS showed suppression of KDM3C expression Knockout of KDM3C in cells exposed to LPS by *P. gingivalis* led to an elevated level of TNF-α, IL-1β, IL-6 and NF-κB signalling.
Francis et al. 2019	H3K4me3, H3K9me3 and H3K27me3 (Chip-polymerase chain reaction)	PDL stem cells	Not reported	Not reported	In PDL stem cells exposed to *P. gingivalis* LPS, the expression of H3K27me3 increased on extracellular matrix and osteogenesis lineage gene promoters, and H3K4me3 expression increased on the promoters of inflammatory response genes.
Kirkpatrick et al., 2018	KDM4B and KDM4E (RT-qPCR)	Connective tissue	Not reported	Periodontitis: at least 1 site with PD > 4 mm, GI 1–3, and PI 1–3. Healthy: PD ≤ 4 mm, GI ≤ 1, and PI ≤ 2.	A significant increase in KDM4B and KDM4E was observed in periodontally diseased vs. healthy tissue.
Li et al., 2018	*HDAC9* (RT-qPCR)	PDL stem cells	Twenty teeth of periodontitis patients were collected from the teeth extracted due to clinical diagnosed chronic periodontitis.	Not reported	*HDAC9* RNA expression levels were significantly increased in human periodontitis PDL stem cells, and TNF-α stimulated healthy PDL stem cells, and their osteogenic capacity was impaired in inflammatory conditions.
Cantley et al., 2016	*HDACs1 to 10* (qPCR, Immunohistochemical detection of *HDAC1, 5, 8 and 9*, Semiquantitative analysis of immunohistochemistry)	Gingival biopsies	For mRNA analysis using real-time PCR, nine samples of periodontitis and eight samples of non-periodontitis tissues were analysed. For immunohistochemical staining, 15 samples of periodontitis tissues and seven samples of non-periodontitis tissues were analysed for both HDAC 1 and 9 staining. For HDAC 5 and 8 staining, eight samples of periodontitis tissues and six non-periodontitis tissues were analysed.	Periodontitis: histologically and radiographic evidence of bone loss Healthy: no or only mild levels of inflammation and had no evidence of bone loss.	mRNA expression of Class I *HDAC1* and *8* and Class II *HDAC5* and *9* were significantly higher in chronic periodontitis samples compared with non-periodontitis samples. There was a twofold increase in *HDAC1, 8 and 5* expression and a threefold increase in *HDAC9* expression in chronic periodontitis samples compared with their counterpart. *HDAC1* was expressed by CD3 and TNF-α positive inflammatory cells in periodontitis tissues.
Martins et al., 2016	Histone H3	Oral epithelial cells	Not reported	Not reported	Abrupt but short-lived increased acetylation of H3 induced by LPS from *P. gingivalis* and heated inactivated *F. nucleatum*. *E. coli* LPS resulted in a delayed but powerful induction of histone acetylation compared with *P. gingivalis* and *F. nucleatum*.
Li et al., 2016	Histone acetyltransferase GCN5 (RT-PCR)	PDL stem cells	Healthy (n = 5), periodontitis (n = 5)	Healthy: absence of BoP, PD < 4 mm and CAL< 3 mm. Periodontitis: alveolar bone loss (2/3) and more than 1 pocket (depth 5 mm)	Inflammation in the microenvironment resulted in downregulation of GCN5 expression, leading to decreased osteogenic differentiation of PDL stem cells.
Yin et al., 2011	*HDAC1* and *HDAC2*	Gingival epithelial cells	Not reported	Not reported	The gene expression of both *HDAC1* and *HDAC2* significantly decreased in cells treated with *P. gingivalis* and *F. nucleatum*, compared with unstimulated controls after 24 h.
	Histone H3K4me3	Gingival epithelial cells			The protein amount was significantly decreased when cells were stimulated with *P. gingivalis* compared with unstimulated controls, but not with *F. nucleatum*.
	*HDAC1* and *HDAC2* (qRT-PCR)	Immortalised keratinocyte cell line			The gene expression of *HDAC1* was significantly decreased in cells treated with *P. gingivalis* after 24 h, but not with *F. nucleatum*. The gene expression of *HDAC2* slightly increased with *F. nucleatum* and *P. gingivalis* initially, then decreased after 24 h.

Abbreviations: CD3, cluster of differentiation 3; HDAC, histone deacetylases; IL, interleukin; KDM, lysine-specific demethylase; LPS, lipopolysaccharide; NF-κB, nuclear factor kappa B; PDL, periodontal ligament; TNF-α, tumour necrosis factor alpha.

**Table 3:** Summary of selective publications included for analysis of histone deacetylase inhibitors for treating periodontitis.

Author/Year	Type of HDAC Inhibition	Tissue/Cell Type	Case Definitions	Epigenetic Events and Conclusions
Algate et al., 2020	Selective Class I and II HDACs	Monocytic cells Osteoclast progenitors	Not reported	When monocytes were stimulated with TNF, HDAC-1i and HDAC-2i both reduced the mRNA expression of pro-inflammatory cytokines IL-1β and TNF and chemokines MIP-1α and MCP-1 in a dose-dependent manner. TNF-stimulated osteoclasts were sensitive to HDAC-1i, reducing the no. and size of osteoclastic cells in vitro. Both HDAC-1i and HDAC-2i diminished osteoclast activity. Both HDAC-1i and HDAC-2i reduced the mRNA expression of the osteoclast signalling factors TRAF-6, TRAP, Cath K and DC-STAMP. No effect was found when inhibiting Class II HDACs (*HDAC5* and *HDAC7*).
Lagosz et al., 2020	Combined and selective Class I and II HDACs	Gingival fibroblasts	Not reported	Treatment of healthy gingival fibroblasts with the pan-HDACi or HDAC3/6i prior to *P. gingivalis* infection significantly reduced the induction of several inflammatory mediators (CCL2, CCL5, CXCL10, IL1B, COX2 and MMP3) that contribute to periodontitis pathogenesis. Treatment of periodontally diseased gingival fibroblasts with HDAC3/6i suppressed the induction of CCL2, CCL5, CXCL10, IL1B, MMP3 and MMP1 to a similar degree as cells from healthy individuals.
Li et al., 2018	HDAC9 (RT-PCR)	PDL stem cells	Not reported	Downregulation of HDAC9 by HDAC inhibitors rescued the osteogenic differentiation capacity of inflammatory PDL stem cells to a similar level with the healthy PDL stem cells.
Huynh et al., 2017	Combined Class I and II HDACs	PDL stem cells	Not reported	Trichostatin A induced acetylated RUNX2, enhancing mineral deposition and the osteogenic potential of PDL stem cells. In vivo bone regeneration of mouse calvaria defects was also significantly enhanced by Trichostatin A pre-treated PDL stem cells
Huynh et al., 2016	Combined Class I and II HDACs (RT-PCR)	PDL stem cells	Not reported	In the presence of the HDAC inhibitor Trichostatin A osteogenic differentiation was induced and osteoblast-related gene expression was increased significantly (without any adipogenic differentiation). Alkaline phosphatase activity and mineral nodule formation were also enhanced.
Cantley et al., 2011	Combined Class I and II HDACs (Real-time PCR analysis)	Alveolar bone and soft tissue	Not reported	Periodontitis-induced mice treated with HDACi 1179.4b exhibited a significant reduction in osteoclast numbers and suppression of bone loss, although no suppression of inflammation. Periodontitis-induced mice treated with HDACi MS-275 had little effect on bone loss but had a non-significant reduction in inflammation.
Cantley et al., 2011	Combined Class I and II HDACs (Real-time PCR analysis)	Monocytic cells Osteoclast progenitors	Not reported	During the late stages of osteoclast formation, 1179.4b significantly reduced the expression of osteoclast transcription factors NFATc1 and OSCAR. Furthermore, 1179.4b almost completely inhibited TRAP expression and osteoclast resorption, reducing the no. of pits formed in a dose-dependent manner. This was confirmed with a 13-fold reduction in CTR gene expression (osteoclast gene associated with ability to resorb bone) corresponding with a marked inhibition of resorption.

Abbreviations: Cath K, cathepsin K; CCL, chemokine ligand; CTR, calcitonin receptor; DC-STAMP, Dendritic Cell-Specific Transmembrane Protein; HDAC, histone deacetylases; HDACi, histone deacetylase inhibitors; IL, interleukin; KDM, lysine-specific demethylase; LPS, MCP-1, monocyte chemoattractant protein-1; MIP-1α, macrophage inflammatory protein 1 alpha; MMP-3, matrix metalloproteinase; NFATC1, Nuclear factor of activated T-cells, cytoplasmic 1; OSCAR, osteoclast-associated receptor; TNF, tumour necrosis factor; TRAF6, TNF Receptor Associated Factor 6; TRAP, Triiodothyronine Receptor Auxiliary Protein.

## Data Availability

Not applicable.
